# Control of PTH secretion by the TRPC1 ion channel

**DOI:** 10.1172/jci.insight.132496

**Published:** 2020-04-23

**Authors:** Marta Onopiuk, Bonnie Eby, Vasyl Nesin, Peter Ngo, Megan Lerner, Caroline M. Gorvin, Victoria J. Stokes, Rajesh V. Thakker, Maria Luisa Brandi, Wenhan Chang, Mary Beth Humphrey, Leonidas Tsiokas, Kai Lau

**Affiliations:** 1 Department of Cell Biology and; 2 Department of Medicine, Division of Nephrology, University of Oklahoma Health Sciences Center, Oklahoma City, Oklahoma, USA.; 3 Department of Surgery, Oklahoma City, Oklahoma, USA.; 4 Academic Endocrine Unit, Radcliffe Department of Medicine, University of Oxford, Oxford, United Kingdom.; 5 Department of Biomedicals Sperimentals and Clinicals Sciences, Università degli Studi di Firenze and Fondazione FIRMO, Florence, Italy.; 6 Endocrinology and Metabolism, Department of Medicine, UCSF, San Francisco, California, USA.; 7 Department of Medicine, Division of Rheumatology, Immunology, and Allergy, University of Oklahoma Health Sciences Center, Oklahoma City, Oklahoma, USA.; 8 Department of Veterans Affairs, Oklahoma City, Oklahoma, USA.

**Keywords:** Cell Biology, Endocrinology, Calcium

## Abstract

Familial hypocalciuric hypercalcemia (FHH) is a genetic condition associated with hypocalciuria, hypercalcemia, and, in some cases, inappropriately high levels of circulating parathyroid hormone (PTH). FHH is associated with inactivating mutations in the gene encoding the Ca^2+^-sensing receptor (*CaSR*), a GPCR, and *GNA11* encoding G protein subunit α 11 (Gα11), implicating defective GPCR signaling as the root pathophysiology for FHH. However, the downstream mechanism by which CaSR activation inhibits PTH production/secretion is incompletely understood. Here, we show that mice lacking the transient receptor potential canonical channel 1 (TRPC1) develop chronic hypercalcemia, hypocalciuria, and elevated PTH levels, mimicking human FHH. Ex vivo and in vitro studies revealed that TRPC1 serves a necessary and sufficient mediator to suppress PTH secretion from parathyroid glands (PTGs) downstream of CaSR in response to high extracellular Ca^2+^ concentration. Gα11 physically interacted with both the N- and C-termini of TRPC1 and enhanced CaSR-induced TRPC1 activity in transfected cells. These data identify TRPC1-mediated Ca^2+^ signaling as an essential component of the cellular apparatus controlling PTH secretion in the PTG downstream of CaSR.

## Graphical abstract

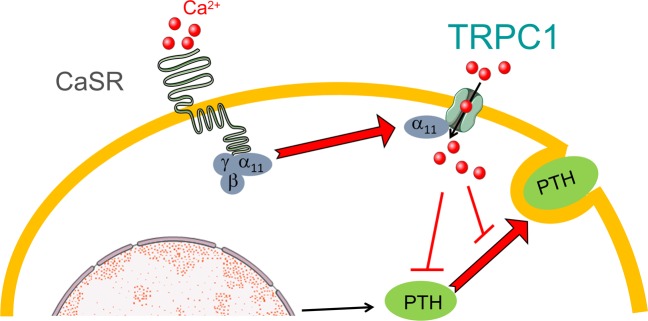

## Introduction

Parathyroid hormone (PTH) is a critical hormone for Ca^2+^ homeostasis. In normal conditions, levels of PTH are tightly controlled by serum Ca^2+^ via a negative feedback mechanism in which high serum Ca^2+^ levels suppress the production and/or secretion of PTH from the parathyroid gland (PTG). This feedback mechanism prevents the development of hypercalcemia or hypocalcemia. In primary hyperparathyroidism often resulting from PTG adenomas, abnormally high levels of PTH result in hypercalcemia, whereas in secondary hyperparathyroidism, frequently seen in renal failure, elevated PTH levels are actually associated with hypocalcemia. Patients with naturally occurring mutations in the gene encoding the Ca^2+^-sensing receptor (*CaSR*) (1, 2), *GNA11* encoding G protein subunit α 11 (Gα11) (3), or *AP2S1* encoding the clathrin-associated adaptor protein-2 σ subunit 2 (AP2σ2) (4) develop familial hypocalciuric hypercalcemia (FHH), characterized by hypercalcemia and hypocalciuria and, in some cases, by inappropriately elevated levels (5). Proteins encoded by the FHH-associated genes function in a linear signaling pathway within the PTG to suppress production and secretion of PTH in response to hypercalcemia. However, an outstanding question has been how hypercalcemia causes the suppression of PTH production and secretion. It has been suggested that a critical step involves a transient rise in intracellular Ca^2+^ concentration in response to high serum Ca^2+^ levels (6, 7). In contrast to most endocrine systems, where an increase in intracellular Ca^2+^ concentration triggers exocytosis and hormonal release, the PTG is unique in that an increase in intracellular Ca^2+^ concentration instead suppresses PTH release (7) The molecular identity of the ion channels mediating this transient increase in intracellular Ca^2+^ concentration driving the suppression of PTH release has been unknown. Therefore, identification of these channels and the mechanisms underlying CaSR-induced Ca^2+^ signaling suppression of PTH secretion are of paramount importance in understanding and treating pathological conditions resulting from abnormal PTH levels.

CaSR is coupled to 3 subclasses of G proteins, Gα11/αq, Gαi/o, and Gα12/13 (8, 9). The identification of *GNA11* as an FHH-associated gene and further functional and genetic studies on Gα11 indicate that the coupling of CaSR to Gα11 may be directly linked to the suppression of PTH production and/or secretion. Activation of Gα11/αq-coupled CaSR leads to the generation of inositol trisphosphate (IP_3_), which triggers Ca^2+^ release from ER and stimulation of Ca^2+^ influx from the extracellular fluid mediated by store- and/or receptor-operated Ca^2+^ entry (SOCE and/or ROCE) channels, localized at the plasma membrane. SOCE channels are defined as the channels activated exclusively via the depletion of the intracellular Ca^2+^ stores following the activation of any pathway that involves the generation of IP_3_ as a second messenger and involve the ER Ca^2+^ sensor, stromal interaction molecule 1 (STIM1), and the pore-forming subunit, Orai1 (10). ROCE channels are activated via intermediaries generated during the activation of cell surface receptors, and nearly all of the 28 known TRP channels can function as ROCE channels, depending on cell and tissue contexts (11, 12). Therefore, theoretically both SOCE and ROCE channels can be activated following the stimulation of the CaSR. Transient receptor potential canonical channel 1 (TRPC1) belongs to the canonical subgroup of TRP channels and has been implicated in both SOCE and ROCE (13). TRPC1 can be activated by CaSR in rabbit mesenteric arteries (14), colonic epithelial (15, 16), endothelial (17), and breast cancer cells (18), making it an ideal candidate for mediating the suppression of PTH secretion downstream of CaSR in the PTG.

Using a combination of in vivo, ex vivo, and in vitro approaches, our studies show that TRPC1 functions downstream of CaSR in the suppression of PTH secretion and that *Trpc1*-null mice show an FHH-like phenotype. However, its mechanism of activation does not involve store depletion but instead involves a protein-protein interaction with Gα11, a protein directly coupled to CaSR and genetically implicated in FHH. Thus, our study identifies TRPC1 as one of the ion channels mediating the mechanism by which high Ca^2+^ serum levels suppress PTH secretion.

## Results

### Trpc1^–/–^ mice develop FHH.

**Figure 1:**
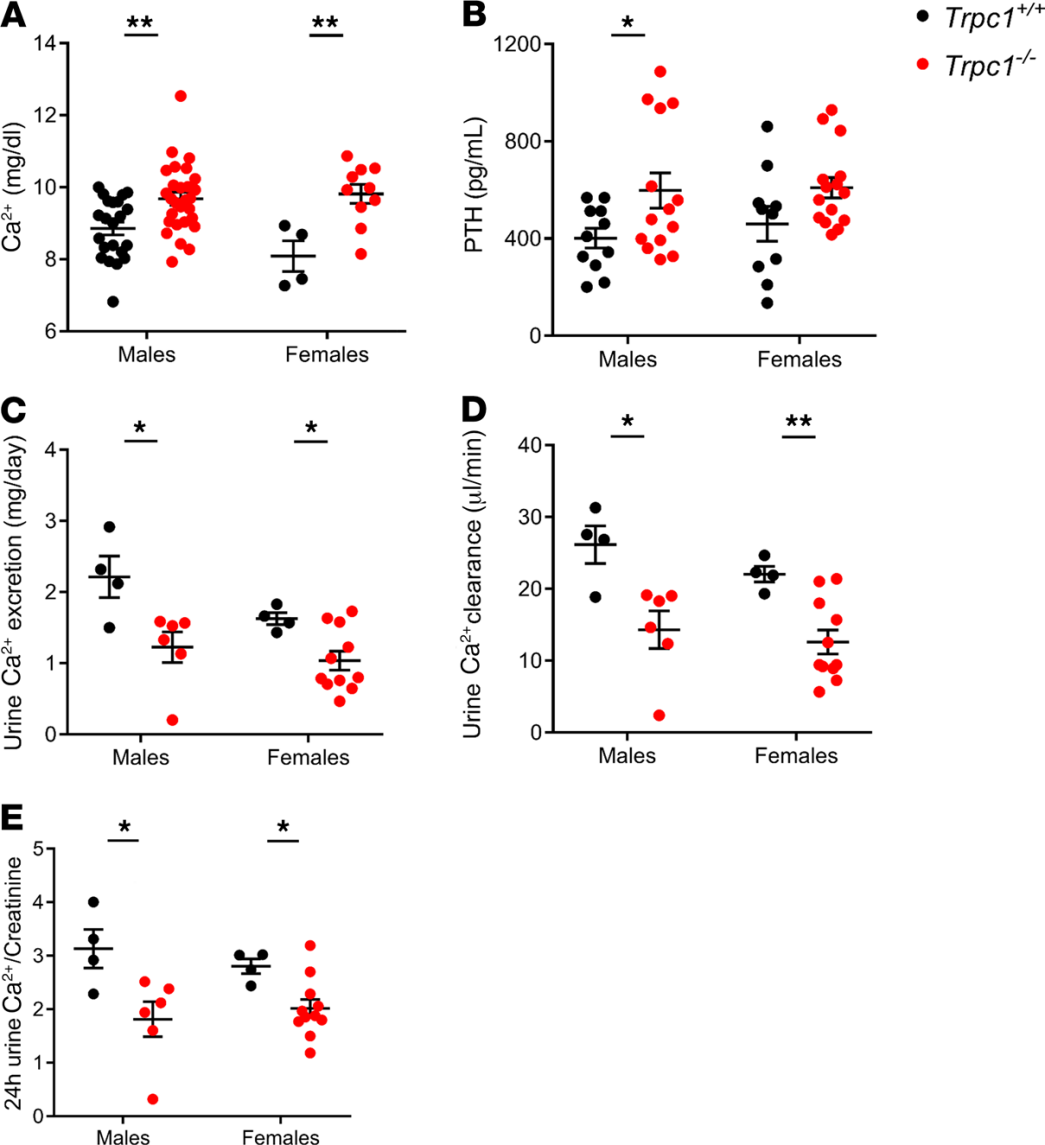
*Trpc1*^–/–^ mice exhibit hypercalcemia, hyperparathyroidism, and hypocalciuria. (**A**) Serum Ca^2+^ levels (mg/dl) in 7-month-old *Trpc1^+/+^* and *Trpc1^–/–^* fasted males and females. **P < 0.01, Student’s *t* test. (**B**) Serum PTH levels (pg/ml) in 7-month-old *Trpc1*^+/+^ and *Trpc1^–/–^* fasted males and females. *P < 0.05, Student’s *t* test. (**C**) Urine Ca^2+^ excretion (mg/day) in 7-month-old *Trpc1^+/+^* and *Trpc1^–/–^* males and females. *P < 0.05, Student’s *t* test. (**D**) Ca^2+^ clearance (μl/min) in 7-month-old *Trpc1^+/+^* and *Trpc1^–/–^* males and females. *P < 0.05; **P < 0.01, Student’s *t* test. (**E**) Twenty-four-hour urine Ca^2+^/creatinine ratio in 7-month-old *Trpc1^+/+^* and *Trpc1^–/–^* males and females. *P < 0.05, Student’s *t* test.

Global *Trpc1^–/–^* mice have been described previously (19). Homeostatic serum Ca^2+^ levels were significantly higher in male and female *Trpc1^–/–^* mice at 7 months of age (Figure 1A). Hypercalcemia was observed at early as 3.5 months and persisted up to 21.5 months in age-matched null littermate males (11.31 ± 0.28 mg/dl in *Trpc1^–/–^* vs. 9.92 ± 0.41 mg/dl in *Trpc1^+/+^*, *P* < 0.05) (Supplemental Figure 1A; supplemental material available online with this article; https://doi.org/10.1172/jci.insight.132496DS1). *Trpc1^+/-^* male mice exhibited significant hypercalcemia (Supplemental Figure 1B), suggesting that heterozygous deletion of *Trpc1* was sufficient to produce a hypercalcemic phenotype. PTH levels were significantly higher in 7-month-old homozygous mutant males and inappropriately high in 7-month-old homozygous mutant females (because they were not suppressed by hypercalcemia as expected in normal physiology) (Figure 1B). Inappropriately high PTH levels were observed in younger and older mice (Supplemental Figure 1C). Hypercalcemia was present in either fasted in *Trpc1^–/–^* mutant male or female mice (10 hours prior to blood collection) or animals allowed to feed ad libitum, arguing against increased gut Ca^2+^ absorption as the mechanism for the hypercalcemia in mice lacking *Trpc1* (Supplemental Figure 1D). Despite hypercalcemia, 24-hour urine Ca^2+^ excretion (Figure 1C), renal Ca^2+^ clearance (Figure 1D), and the urine Ca^2+^/creatinine ratio were all reduced in 7-month-old *Trpc1^–/–^* males and females (Figure 1E). Reduced urine Ca^2+^ clearance persisted up to 21.5 months (5.8 ± 3.4 μl/min in *Trpc1^–/–^* males vs. 29.3 ± 7.9 μl/min in *Trpc1^+/+^* males). Serum Mg^2+^, renal Mg^2+^ clearance, urine Mg^2+^ excretion, and the 24-hour urine Mg^2+^/creatinine ratio were unaffected by deletion of *Trpc1* (Table 1). These data indicate that *Trpc1^–/–^* mice are hypocalciuric and that, overall, they show the classic triad of FHH of hypocalciuria, hypercalcemia, and significantly elevated or inappropriately high levels of circulating PTH.

To evaluate if these phenotypes were secondary to renal disease and/or disorders of vitamin D metabolism, we measured serum levels of 1,25 (OH)_2_ vitamin D, creatinine, and calcitonin. 1,25 (OH)_2_ vitamin D was unchanged in 7-month-old males (Table 1). Similarly, serum creatinine in Trpc1^–/–^ mice was similar to that in WT mice at 1 year of age (Table 1). Creatinine clearance in *Trpc1^–/–^* mice was comparable to that in WT mice (Table 1), indicating that renal failure could not have accounted for the hypercalcemia and elevated PTH levels seen in *Trpc1^–/–^* mice. Serum phosphorus levels in *Trpc1^–/–^* mice were similar to those in *Trpc1^+/+^* mice (Table 1). Hematocrits were not different at 8.5 months of age and were lower at 10.5 and 21.5 months of age, showing that hemoconcentration was not responsible for the hypercalcemia (Table 1). Plasma levels of calcitonin, another regulator of serum Ca^2+^, were unchanged in 7-month-old *Trpc1*-null males (Table 1), suggesting that deletion of *Trpc1* does not affect calcitonin secretion from C cells of the thyroid gland that could indirectly influence serum Ca^2+^ and/or PTH secretion.

Despite high PTH levels seen in primary hyperparathyroidism typically being associated with low bone mass and osteoporosis, patients with FHH manifest slightly reduced, normal, or even increased bone mass (20, 21). Mildly increased bone mass is also seen in mice lacking the CaSR in the PTG (22). Our previous work has shown that 12-week-old male *Trpc1^–/–^* mice have slightly increased bone mass (23). We extended the analysis to 19-month-old animals and found that *Trpc1^–/–^* mice had 83% increase in bone volume to tissue volume, 27% reduction in trabecular spacing, 36% increase in trabecular number and 181% increase in connectivity density (Supplemental Figure 2, A–D).

### TRPC1 mutations not detected in patients with FHH.

The phenotype of the *Trpc1^–/–^* mice is consistent with that of FHH in humans, and the possibility that mutations in the *TRPC1* gene may be a cause of hypercalcemia in some patients with FHH who did not have *CASR*, *GNA11*, or *AP2S1* mutations was therefore explored. Sanger DNA sequence analysis (14 patients) or whole exome sequencing (5 patients) did not detect any point mutations, deletions, insertions, or unreported SNPs. However, a 16-bp segment corresponding to the 5′UTR in short form of TRPC1 or amino acid residues VGAGG in long form of TRPC1 (Supplemental Figure 3) could not be reliably ascertained due to repetitive elements. A binomial analysis predicted that the use of 19 samples in total would have a greater than 95% and 98% likelihood of detecting at least 1 TRPC1 mutation, assuming a mutation prevalence of 15% and 20%, respectively (24). Whole exome sequencing did identify a common polymorphism (p.A14T, present in 7490 of 261,160 alleles in the gnomAD v2.1.1 database) in 1 patient with FHH.

### TRPC1 is required for the suppression of PTH secretion in isolated mouse PTGs.

**Figure 2:**
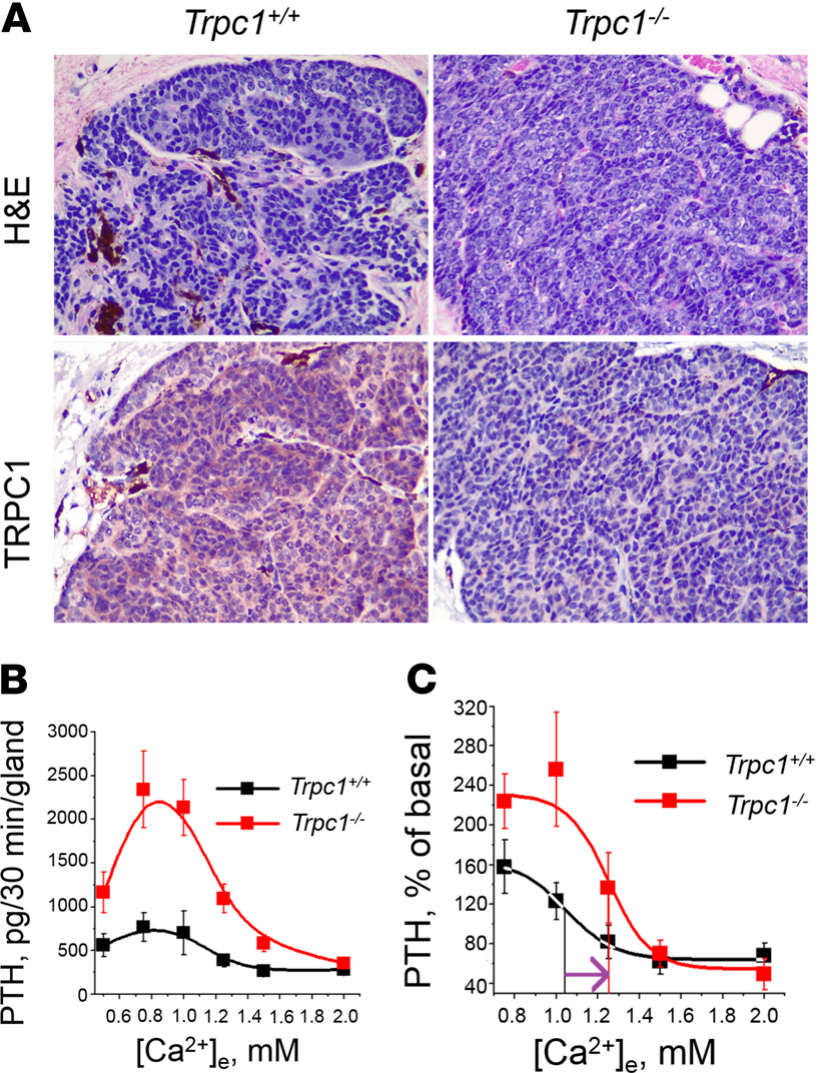
Parathyroid glands lacking TRPC1 fail to properly control PTH secretion. (**A**) TRPC1 is widely expressed in normal mouse PTGs. Top: H&E staining of *Trpc1^+/+^* (left) and *Trpc1^–/–^* (right) glands. Bottom: Expression of TRPC1 in WT (left) and *Trpc1*-null (right) PTGs by immunohistochemistry using a mouse monoclonal antibody against TRPC1 (1F1). Original magnification, ×40. (**B**) Responses of PTH release at different [Ca^2+^]_e_ (pg/30 min/gland) in PTGs isolated from 8 (3 males and 5 females) WT and 8 (3 males and 5 females) 14-week-old *Trpc1*-null mice. (**C**) Ca^2+^ dose-response curves shown in **B** were normalized and are expressed as a percentage of the PTH release at 0.5 mM Ca^2+^. Perpendicular lines depict Ca^2+^ set points (1.04 mM ± 0.15 for *Trpc1^+/+^* and 1.25 mM ± 0.08 for *Trpc1^–/–^* mice).

TRPC1 was widely expressed in WT but not in mutant PTGs (Figure 2A). To test for a PTG-autonomous effect of TRPC1 on PTH secretion, we determined the secretory capacity of isolated PTGs from groups of both 14-week-old male and female mice, as was done previously for the CaSR (22). Male and female mice had similar PTH secretion maximum (Rmax) and minimum (Rmin) as well as Ca^2+^ set points; therefore, we pooled the data to increase the power of the analysis. Absolute Rmax values were approximately 3-fold higher in *Trpc1^–/–^* PTGs compared with those in *Trpc1*^+/+^ PTGs (Figure 2B), accompanied by a significant (*P* < 0.05) rightward shift in the Ca^2+^ set point from 1.04 ± 0.15 mM in *Trpc1^+/+^* mice to 1.25 ± 0.08 mM in *Trpc1^–/–^* mice (Figure 2C). These ex vivo effects were more severe than the deletion of 1 allele of *Casr* in the PTGs of 3-month-old mice but less severe than the deletion of 2 *Casr* alleles (22, 25), suggesting that TRPC1 may significantly contribute (by more than 50%) to the effects of CaSR in the secretion of PTH from the isolated PTG.

### Parathyroid cells depleted of TRPC1 secrete more PTH.

**Figure 3:**
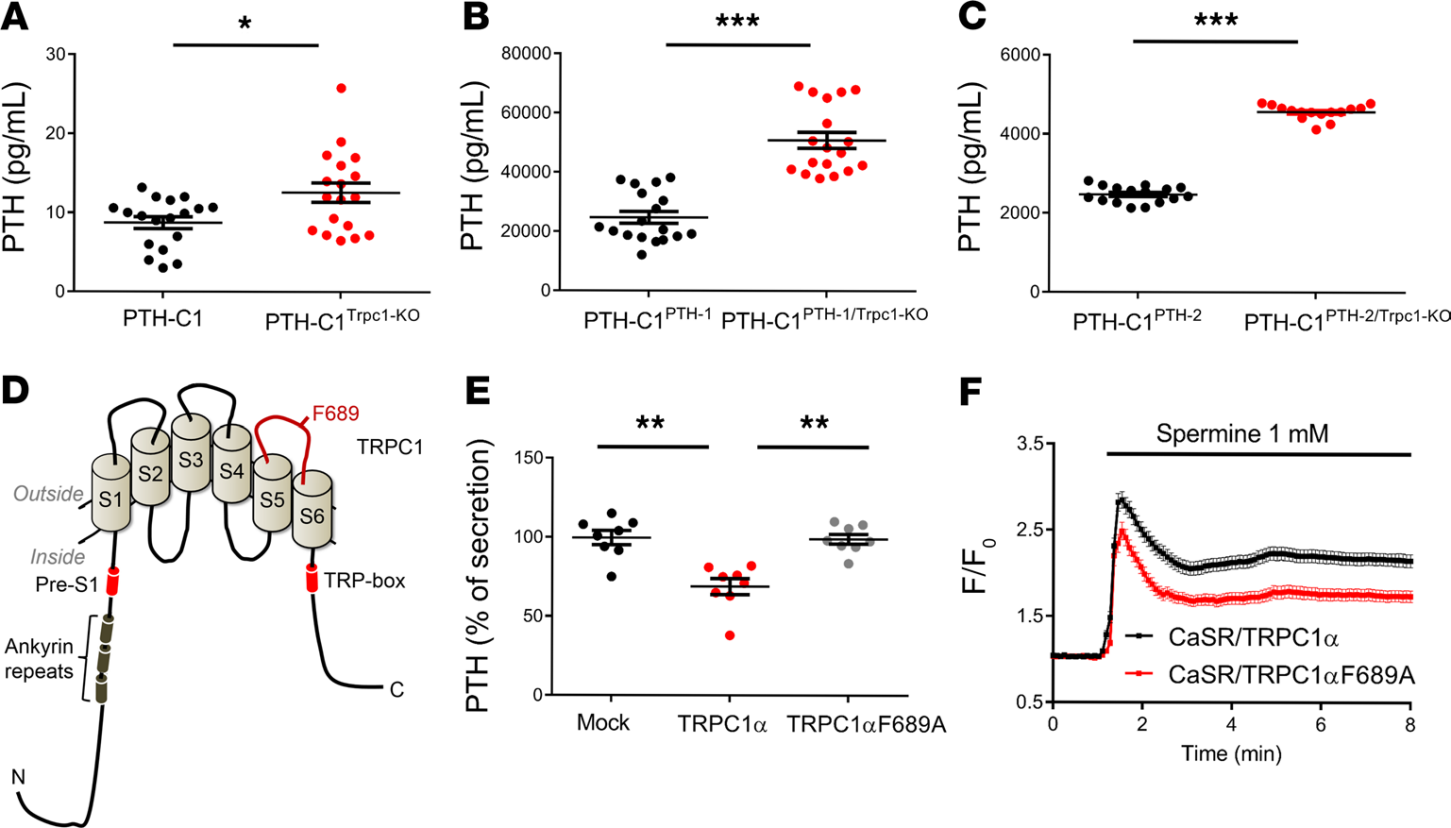
Inactivation of the *Trpc1* gene increases PTH secretion. (**A–C**) Absolute PTH levels in media of WT PTH-C1 cells or cells lacking TRPC1 in the presence or absence of exogenously transfected PTH. *P < 0.05; ***P < 0.001, Student’s *t* test. (**D**) TRPC1 topology and location of F689. Pore-forming region connecting S5 and S6 is shown in red. Ankyrin repeats are shown as dark green cylinders and pre-S1 and TRP-box domains as shown are red cylinders. (**E**) Readdition of WT mouse TRPC1α but not the TRPC1αF689A pore mutant rescues suppressed PTH secretion in cells lacking endogenous rat TRPC1 (PTH-C1^PTH-2/Trpc1-KO^) cells. Data from 9–18 measurements were pooled from 3–6 independent experiments. **P < 0.01, 1-way ANOVA. (**F**) Spermine-induced changes in free intracellular Ca^2+^ in HEK293 cells transiently cotransfected with CaSR plus WT TRPC1α (black, *n* = 296 cells pooled from 3 experiments) or TRPC1α-F689A (red, *n* = 276 cells pooled from 3 experiments).

Derived from rat PTG, PTH-C1 cells express CaSR, produce and secrete PTH, and are currently the only known cell line available for studying PTH secretion in rodents (26). Using CRISPR/Cas9 gene editing, we introduced a frameshift in the *Trpc1* locus in PTH-C1 cells and generated several stable clones. One of these clones, PTH-C1**^Trpc1-KO^ (Supplemental Figure 4, A and D), was used for functional PTH secretion assays. Deletion of TRPC1 led to a 44% increase in secreted PTH (from 8.7 ± 0.8 pg/ml in WT cells to 12.6 ± 1.2 pg/ml in PTH-C1**^Trpc1-KO^ cells) in the presence of low extracellular Ca^2+^ concentration (0.5 mM Ca^2+^), which should promote PTH secretion (Figure 3A). Similar effects were seen when PTH-C1 cells were treated with Pico145, a specific inhibitor of TRPC1/TRPC4/TRPC4 channels (27) (Supplemental Figure 5A). RT-PCR failed to detect TRPC4 or TRPC5 mRNAs in PTH-C1 cells, indicating that Pico145 increased PTH secretion, most likely by inhibiting endogenous TRPC1 in these cells (Supplemental Figure 5B).

To examine whether endogenous TRPC1 controls PTH secretion rather than gene expression, we generated and characterized stable clones overexpressing mouse PTH using a heterologous promoter (CMV) that should not be affected by TRPC1 levels. Two individual clones highly expressing PTH (PTH-C1**^Pth-1^ ~20,000-fold and PTH-C1**^Pth-2^ ~2,000-fold compared with parental PTH-C1 cells) were subsequently transfected with a TRPC1-specific CRISPR/Cas9 construct, and two individual clones (PTH-C1**^Pth-1/*Trpc1*-KO^ and PTH-C1**^Pth-2/*Trpc1*-KO^) with complete deletion of TRPC1 (verified by Sanger sequencing of the recombination site at the *Trpc1* locus, Supplemental Figure 4, B and C) were used for functional assays. PTH-C1**^Pth-1*/Trpc1*-KO^ cells secrete 50.9 ± 2.7 ng/ml PTH, which was 106% higher than that of PTH-C1**^Pth-1^ cells (24.8 ± 2.0 ng/ml) (Figure 3B). Similarly, PTH-C1**^Pth-2/*Trpc1*-KO^ cells showed 85% higher PTH secretion (4574 ± 48 pg/ml) compared with PTH-C1**^Pth-2^ cells (2473 ± 53 pg/ml) (Figure 3C). Transfecting back WT TRPC1α suppressed PTH secretion by 31% in PTH-C1**^Pth-2/*Trpc1*-KO^ cells (Figure 3E). However, transfection of TRPC1α F689A, with a single amino acid mutation in its pore region (Figure 3D) that reduces Ca^2+^ permeability (28) and CaSR-induced Ca^2+^ influx (Figure 3F), failed to suppress PTH secretion (0.7%) in these cells (Figure 3E). In the presence of mutant TRPC1αF689A, spermine activation of CaSR induced less intracellular Ca^2+^ accumulation than PTH-C1 cells coexpressing CaSR and WT TRPC1α (Figure 3F). These data show that TRPC1α has an essential and specific role in suppressing PTH secretion in PTH-C1 cells and this property involves its ability to conduct Ca^2+^.

### TRPC1 overexpression suppresses PTH secretion.

**Figure 4:**
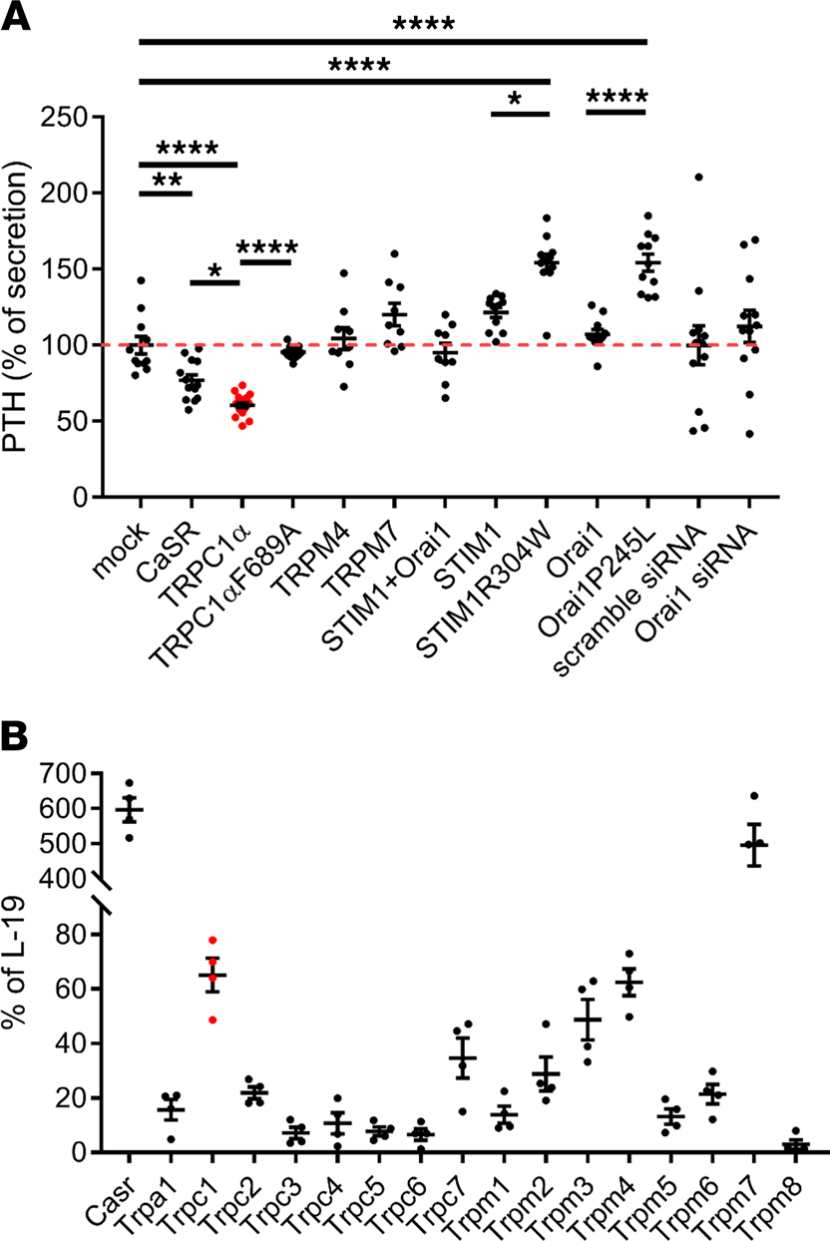
TRPC1 overexpression suppresses PTH secretion independently of SOCE and other TRP channels expressed in the mouse PTG. (**A**) Normalized levels of secreted PTH in media of PTH-C1 cells transiently transfected with indicated expression plasmids or siRNAs (*n* = 9 measurements pooled from 3 independent experiments). *P < 0.05; **P < 0.005; ***P < 0.001, ****P < 0.0001, 1-way ANOVA. (**B**) Expression levels of TRP channel mRNAs in mouse PTG by Affymetrix Mouse Microarray analyses. L-19 was used as the housekeeping gene (*n* = 4 batches of 20 PTGs).

Next, we determined whether TRPC1 overexpression can suppress PTH secretion in PTH-C1 cells. Overexpression of WT TRPC1α, but not TRPC1αF689A, suppressed PTH secretion in PTH-C1 cells (Figure 4A). We also compared side-by-side overexpression of CaSR (positive control) and 2 other highly expressed TRP channels in the PTG, TRPM4 or TRPM7 (Figure 4B), or WT STIM1, Orai1 and their constitutively active mutants, STIM1R304W and Orai1P245L (29). We chose to test the effect of WT and active mutants of STIM1 and Orai1 on PTH secretion because we have previously shown that TRPC1 functions together with these proteins to increase the dynamic range of SOCE channels (23). Overexpression TRPC1α or CaSR suppressed PTH secretion (Figure 4A). Overexpression of TRPM4, TRPM7, STIM1, or Orai1 had no effect on PTH secretion, whereas overexpression of constitutively active STIM1 or Orai1 mutants increased PTH secretion. Knockdown of Orai1 did not affect PTH secretion. The positive effect of constitutively active STIM1/Orai1 mutants on PTH release is consistent with the well-known role of SOCE channels in hormonal release, exocytosis, and mast cell degranulation (30). These data provided additional evidence that TRPC1 has a specific, essential, and sufficient role in suppressing PTH secretion in PTH-C1 cells and does so independently of its ability to enhance SOCE.

### TRPC1 functions downstream of CaSR in PTH-C1 or HEK293 cells.

**Figure 5:**
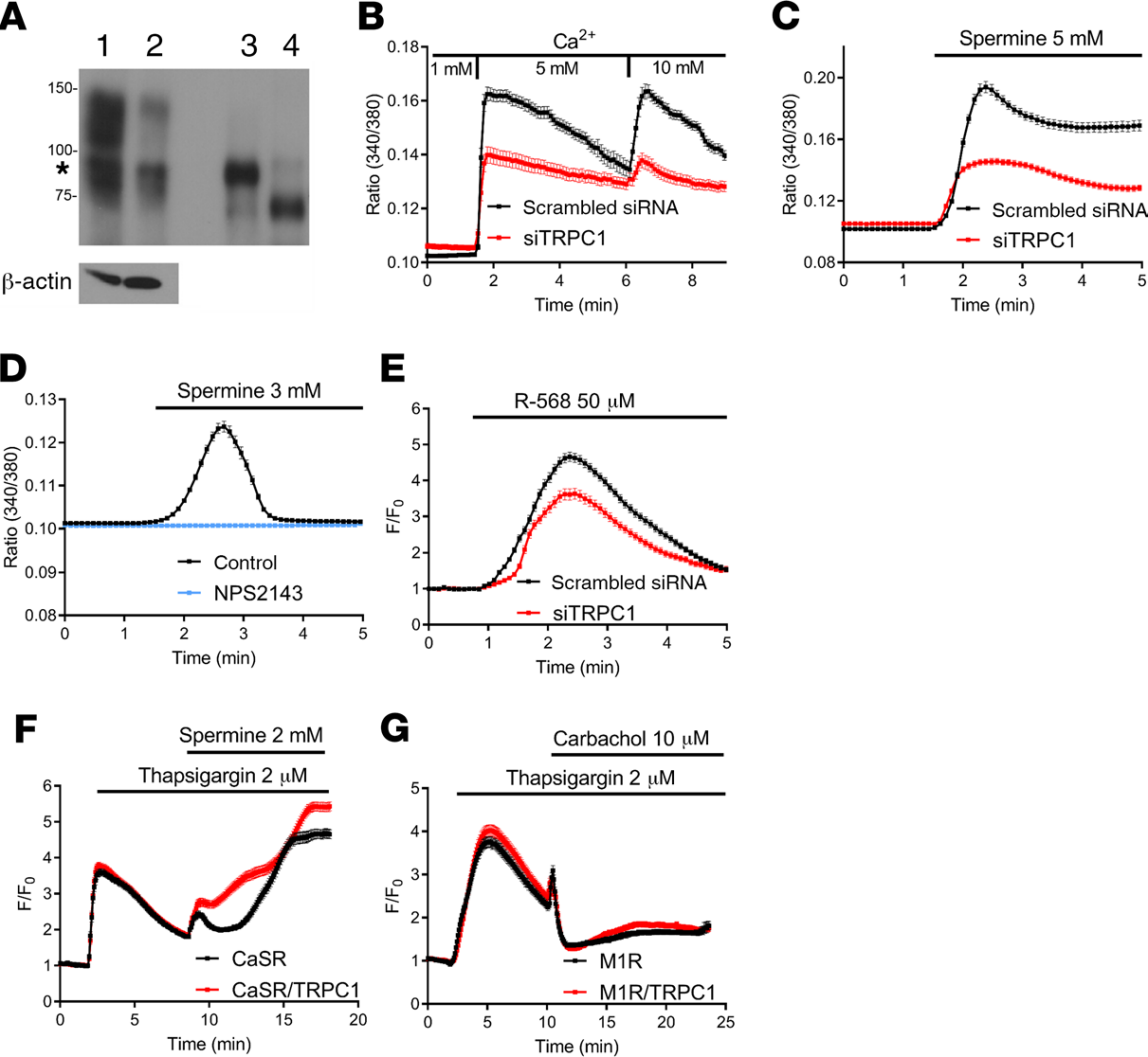
TRPC1 is required for CaSR-induced Ca^2+^ signaling in PTH-C1 cells. (**A**) Efficiency of TRPC1 knockdown using RNAi. PTH-C1 cells were transiently transfected with a scrambled siRNA (lane 1), siTRPC1 (lane 2), expression plasmid (positive control) of a long form of TRPC1α (lane 3), or a short form of TRPC1α (lane 4). TRPC1 was immunoprecipitated and immunoblotted with a TRPC1-specific monoclonal antibody (1F1). PTH-C1 cells express predominantly the long form of TRPC1α (indicated by an asterisk). (**B**) Changes in intracellular Ca^2+^ concentration (expressed as fluorescence ratio 340/380) in PTH-C1 cells transiently transfected with a scrambled siRNA (control, black, *n* = 76 cells pooled from 5 independent experiments) or a *Trpc1*-specific siRNA (siTRPC1, red, *n* = 61 cells pooled from 6 independent experiments) and cultured in 1, 5, or 10 mM extracellular Ca^2+^. (**C**) Time course of spermine-induced (5 mM) intracellular Ca^2+^ concentration in PTH-C1 cells transiently transfected with a scrambled siRNA (control, black, *n* = 144 cells pooled from 4 independent experiments) or a *Trpc1*-specific siRNA (siTRPC1, red, *n* = 178 cells pooled from 8 independent experiments). (**D**) Time course of intracellular Ca^2+^ concentration in PTH-C1 cells cultured in 0 extracellular Ca^2+^ and activated by spermine (3 mM) in the presence (blue, *n* = 279 cells from 6 experiments) or absence of NPS2143 (300 nM) (black, *n* = 233 cells from 5 experiments). (**E**) Time course of R568-induced (50 μM) intracellular Ca^2+^ concentration in PTH-C1 cells transiently cotransfected with GCaMP3 and scrambled siRNA (control, black line, *n* = 216 cells pooled from 9 independent experiments) or TRPC1 siRNA (siTRPC1, red line, *n* = 172 cells pooled from 9 independent experiments) in the presence of 1.8 mM extracellular Ca^2+^ concentration. F_0_ was the average fluorescence for 1 minute prior to the addition of R-568. (**F** and **G**) TRPC1 is specifically coupled to CaSR. HEK293 cells were transiently cotransfected with GCaMP3, CaSR (**F**), or m1 muscarinic acetylcholine receptor (m1 AchR, **G**) in the presence or absence of TRPC1 (red or black traces). Cells were first stimulated with thapsigargin (2 μM) to deplete the internal stores and then with spermine (2 mM, **F**) or carbachol (10 μM, **G**) to activate CaSR or m1 AchR, respectively. Changes in intracellular free Ca^2+^ concentration are reported as F/F_0_. Data were pooled from 6 independent experiments totaling 263 cells transfected with CaSR (black, **F**) and 316 cells transfected with CaSR plus TRPC1 (red, **F**) or from 8 independent experiments totaling 190 cells transfected with m1 AchR (black, **G**) and 173 cells transfected with m1 AchR plus TRPC1 (red, **G**).

To test whether TRPC1 functions downstream of CaSR in Ca^2+^ signaling in PTH-C1 cells, we transiently depleted TRPC1 in PTH-C1 cells using a rat *Trpc1-*specific siRNA. *Trpc1* mRNA translation is initiated by 2 alternative start sites. Translation initiation by an upstream leucine generates the long form of TRPC1, whereas initiation by a downstream methionine generates the short form of TRPC1. The 2 isoforms differ by N-terminal extension of 78 amino acids (23). PTH-C1 cells expressed predominantly the long form of TRPC1, which was downregulated in cells transfected by a rat *Trpc1*-specific siRNA (Figure 5A). Depletion of TRPC1 attenuated intracellular Ca^2+^ signaling in cells activated by extracellular Ca^2+^, spermine, or R-568 (Figure 5, B–E), which are all well-established activators of the CaSR (9). The response to spermine represented specific activation of CaSR, since it was completely blocked in the presence of the CaSR-specific inhibitor, NPS2143 (Figure 5D). These data showed that TRPC1 depletion reduced CaSR-induced Ca^2+^ signaling, consistent with the hypothesis that TRPC1 functions downstream of CaSR activation to increase intracellular Ca^2+^ concentration to suppress PTH secretion. Activation of CaSR can lead to the concurrent activation of both SOCE and ROCE channels. Our functional PTH secretion assays suggested that TRPC1 functions independently of SOCE channels in suppressing PTH secretion. Here, we used a heterologous system to test whether TRPC1 can be specifically coupled to CaSR and function independently of SOCE channels in Ca^2+^ signaling. To test for the contribution of TRPC1 in SOCE, HEK293 cells were transfected with TRPC1 and CaSR or m1 acetylcholine receptor (AchR), which is also coupled to Gα11/Gαq, and stimulated with thapsigargin to deplete internal Ca^2+^ stores followed by direct activation of transfected CaSR or m1 AchR with spermine or carbachol, respectively. Expression of TRPC1 enhanced Ca^2+^ signaling in cells transfected with CaSR (Figure 5F) but not m1 AchR (Figure 5G). In addition, overexpression of TRPC1 did not have an effect on thapsigargin-induced Ca^2+^ signaling. These data suggest that TRPC1 is coupled to CaSR via a mechanism independent of depletion of intracellular Ca^2+^ stores, supporting our data from PTH secretion assays in PTH-C1 cells, in which overexpression or knockdown of STIM1 or Orai1 did not affect PTH secretion.

### Gα11 physically interacts with TRPC1 and increases its activity.

**Figure 6:**
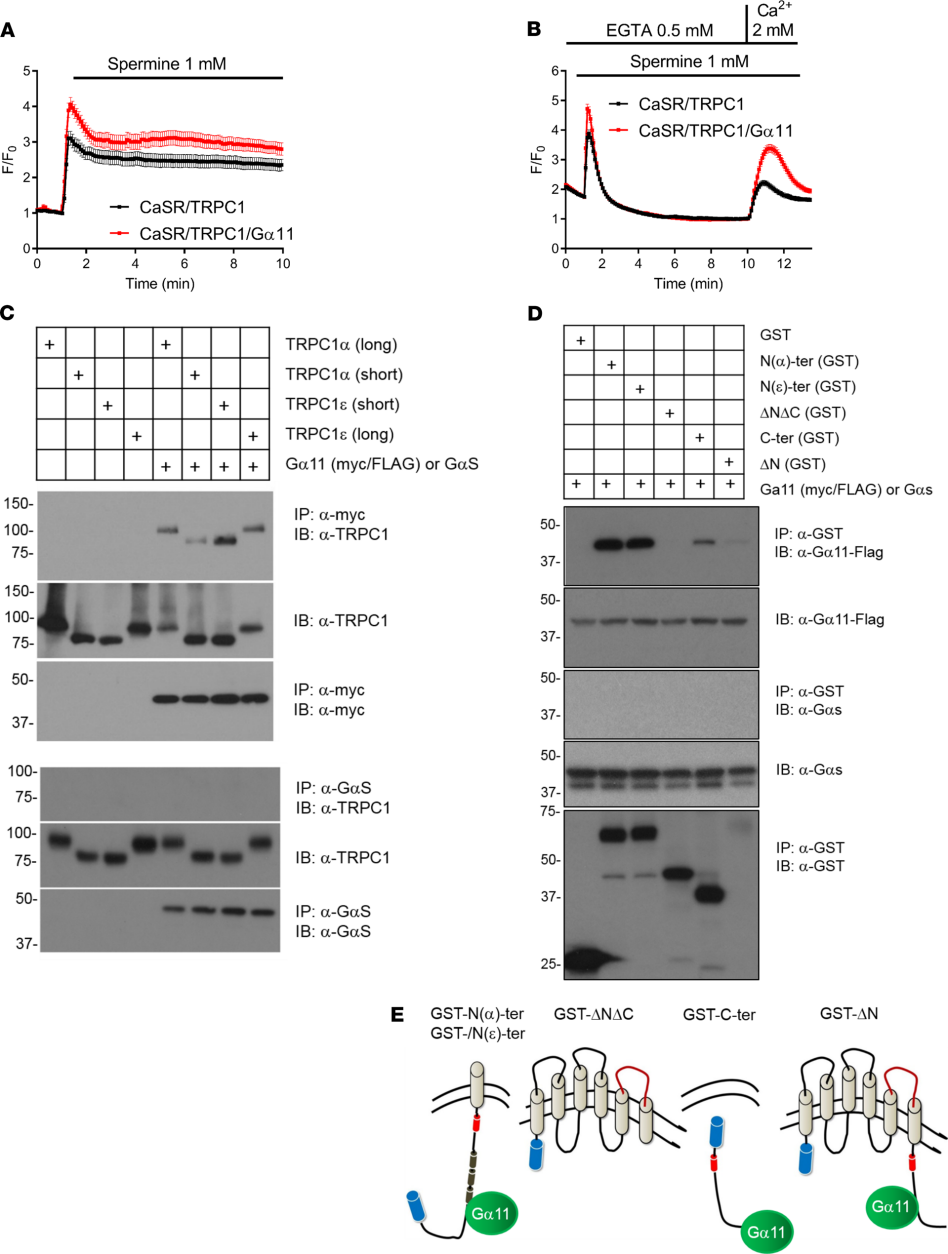
Gα11 physically interacts with TRPC1 and increases its activity. (**A** and **B**) Gα11 increases TRPC1-mediated Ca^2+^ signaling in HEK293 cells. Cells were transiently transfected with CaSR and TRPC1α or CaSR, TRPC1α, and Gα11. Both groups were cotransfected with the fluorescent Ca^2+^ indicator, GCaMP3, and fluorescence intensity was determined by single-cell Ca^2+^ imaging. Changes in intracellular Ca^2+^ concentration (F/F_0_) in response to spermine (1 mM) were determined under physiological conditions (1.8 mM extracellular Ca^2+^, **A**) or in Ca^2+^-free ECS followed by Ca^2+^ readdition (2 mM, **B**). Data were pooled from 74 (black) or 85 (red) cells from 2 independent transfections (**A**) and 318 (black) or 261 (red) cells from 4 independent transfections (**B**). (**C**) HEK293T cells were transiently transfected with the long form of TRPC1ε, short form of TRPC1ε, short form of TRPC1α, long form of TRPC1α, or the same TRPC1 plasmids plus myc-tagged Gα11 (top 3 rows) or GαS (bottom 3 rows). Gα11 was immunoprecipitated using α-myc, and GαS was immunoprecipitated using α-GαS. TRPC1 was detected in the complexes using anti-TRPC1 (top). TRPC1 input is shown in middle panels, and immunoprecipitated Gα11 or GαS is shown in bottom panels. (**D**) Truncation mutants of TRPC1α (GST alone, GST-N(α)-ter, GST-N(ε)-ter, GST-ΔNΔC, GST-C-ter, GST-ΔN) shown in **E** tagged with GST (blue cylinders) were cotransfected with WT Gα11 or GαS. Protein-protein interactions were determined by GST pulldowns followed by immunoblotting. Gα11 (first panel), but not GαS (third panel), interacted with the cytosolic N- or C-tail of TRPC1. Gα11 input is shown in second panel, GαS input is shown in fourth panel, and immunoprecipitated mutants of TRPC1 are shown.

Given the established role of Gα11 in FHH and PTH secretion (3), we asked whether Gα11 overexpression could further enhance CaSR-induced TRPC1-mediated Ca^2+^ influx. Indeed, overexpression of Gα11 augmented TRPC1-mediated Ca^2+^ influx in response to spermine activation of CaSR in HEK293 cells using 2 different protocols (Figure 6, A and B). Gα11 could enhance TRPC1 activity by multiple mechanisms. One possible mechanism could involve complex formation of Gα11 with TRPC1, as previously reported for receptor-activated TRP channels and Gα_q_ (31). Therefore, we examined whether Gα11 could interact with TRPC1. We tested both long and short forms of TRPC1α and TRPC1ε isoforms. TRPC1α is the most widely expressed form of TRPC1, whereas TRPC1ε has a 7–amino acid deletion at the beginning of exon 5 corresponding to the N-terminal cytosolic domain of TRPC1 and, so far, is shown to be expressed in myeloid precursor cells (23). Coimmunoprecipitation experiments in transiently transfected HEK239T cells followed by deletion analysis showed that either TRPC1α or TRPC1ε isoforms interacted with Gα11 through their N- and C-termini (Figure 6, C and D). Consistently, endogenous TRPC1 colocalized with Gα11 in PTH-C1 cells (Supplemental Figure 6). In contrast, TRPC1 did not interact with GαS, showing specificity of TRPC1 to Gα11 (Figure 6, C and D). In light of genetic data in which inactivating mutations in *GNA11* in humans produce a phenotype also seen in *Trpc1*-null mice, plus the molecular and functional data that show that Gα11 coimmunoprecipitates and enhances the activity of TRPC1, we propose that the direct interaction between TRPC1 and Gα11 potentiates the stimulatory effects of CaSR on TRPC1.

## Discussion

The discovery of CaSR had a profound effect on our understanding how PTG cells sense extracellular Ca^2+^ levels (2, 6, 9, 32, 33). However, there are still gaps in our knowledge regarding how PTG cells regulate PTH secretion downstream of CaSR. We provide in vivo, ex vivo, and in vitro evidence that TRPC1 functions downstream of CaSR to suppress PTH secretion. This information can help direct future studies to better our understanding of how the Ca^2+^ signaling can regulate PTH release, calcitonin secretion, and secretion of other hormones from cells with a functional CaSR.

Global deletion of *Trpc1* resulted in a phenotype that showed significant similarities to FHH (21, 34). Statistically, as a group, patients with FHH exhibit mild but significant hypercalcemia, while a few of them show elevated PTH (5, 35), similar to male *Trpc1^–/–^* mice. Hypocalciuria, a hallmark of FHH not typically seen in primary hyperparathyroidism (5, 36), was also observed in our *Trpc1^–/–^* mice. These data are consistent with the idea that TRPC1 functions downstream of CaSR in the PTG and the kidney. Unchanged 1,25 (OH)_2_ vitamin D levels are observed in patients with FHH (34, 37, 38), which is corroborated in our *Trpc1^–/–^* mice. A few differences are noteworthy between *Trpc1^–/–^* mice and patients with FHH. Serum Mg^2+^ is often moderately increased in some patients with FHH (3, 4, 39)*,* but Mg^2+^ levels were normal in *Trpc1^–/–^* mice. Second, *Trpc1^–/–^* mice and mice with kidney-specific deletion of *Casr* have normal serum phosphorous levels (40), unlike patients with FHH with reduced levels (35). Finally, there are changes in skeletal manifestation between our mice and patients with FHH. In FHH, bone mass is generally comparable to that of normal controls (20, 21, 41). However, our *Trpc1*-null mice had substantially increased and progressive changes in bone mass with age. Indeed, previously we reported significantly decreased osteoblast number per bone surface and reduced osteoclast numbers per bone surface in histomorphometric assays of 3-month-old *Trpc1*-null mice (23). These similarities and differences may correlate to compensatory proteins that differ between mice and humans in the kidney, PTG, and bone cells. With the exception of *Casr^+/–^* mice (42) and a newly described mouse model carrying a loss-of-function mutation (D195G) in *Gna11* (43), which recapitulates human FHH both at the disease phenotypic and genetic levels, there are no other suitable mouse models for FHH, including orthologous mouse models. For example, while dominant loss-of-function mutations in *GNA11* result in FHH in patients (3), homozygous global deletion of *Gna11* in the mouse did not produce a significant phenotype (44). The PTG-specific deletion of *Gnaq* combined with global deletion of *Gna11* recapitulated some aspects of FHH in the mouse (45), suggesting potential gene redundancy by *Gnaq* in the mouse but not in humans. Alternatively, dominant negative effects of loss-of-function alleles of *Gna11* could be implicated in FHH. Nevertheless, the generation of additional mouse models of FHH could help advance our understanding of the pathophysiology of FHH and diseases of the PTG. We believe that the global *Trpc1*-KO mouse model presented here can be considered as a suitable mouse model for FHH because it recapitulates several key features of FHH. Additional refinements could be made by the tissue-specific deletion of *Trpc1* in the PTG, bone, and kidney.

Our data suggest that Gα11-mediated signaling downstream of CaSR couples serum Ca^2+^ concentration and PTH secretion. CaSR is linked to several classes of Gα subunits and it remains unknown which subclass(es) and how specific G protein(s) mediate the CaSR’s effects on PTH secretion (9). The identification of naturally occurring mutations in *GNA11* in patients with FHH2 strongly suggests that the Gα11/αq subclass conveys the signal to suppress PTH secretion following activation of CaSR (3). Our data support this hypothesis and further identify the ion channel mediating such an effect. While we favor the idea that TRPC1 is a direct target of activated Gα11, we cannot rule out a possible modulation of TRPC1 activity by other Gα subclasses coupled to CaSR, either directly via protein-protein interactions or indirectly via cAMP/PKA-mediated signaling (Gαi/o) and regulation of the actin cytoskeleton (Gα12/13). The FHH-like phenotype we observed in *Trpc1*-null mice is less severe than the phenotype produced by the deletion of CaSR in mice (22, 42), suggesting that loss of TRPC1 in the PTG produces a milder phenotype than the loss of CaSR signaling. While other Gα11-activated Ca^2+^-permeable channels can work in parallel with TRPC1 to suppress PTH secretion, Ca^2+^-independent signaling could also account for the remaining effect of PTH suppression in *Trpc1*-null cells/tissues. Alternatively, TRPC1-mediated Ca^2+^ signaling may have a permissive role in the suppression of PTH secretion under conditions of abnormally high serum Ca^2+^ levels. Regardless of the exact mechanism by which TRPC1 influences PTH secretion and the extent to which it contributes to the overall effect of CaSR on PTH secretion, our studies identify what is probably the first ion channel that can function downstream of CaSR in the control of PTH secretion and, thus, can spur new studies on the role of Ca^2+^ signaling in PTH homeostasis.

While TRPC1 was the first mammalian TRP channel identified more than 20 years ago (46), its activation mechanism remains unclear (13). Our studies identifying TRPC1 as part of the cellular apparatus controlling PTH secretion have implications in its activation mechanisms. We had shown earlier that TRPC1 can be activated by store depletion (23), but our PTH secretion data in PTH-C1 cells show that activation by store depletion could not account for its effect on PTH secretion. This conclusion is based on PTH secretion data, whereby overexpression or depletion of STIM1 or Orai1 did not affect PTH secretion in PTH-C1 cells and, furthermore, overexpression of constitutively active STIM1 and Orai1 identified in patients with Stormorken or a Stormorken-like syndrome, respectively, enhanced rather than suppressed PTH secretion. While this seems to be at odds with mild hypocalcemia reported in some patients with Stormorken (47), it is in agreement with the well-established role of SOCE channels in exocytosis and hormonal release (30). The hypocalcemia seen in patients with Stormorken could be due to secondary effects of STIM1R304W in tissues other than the PTG, such as the kidney or bone. More studies are necessary to determine the effect of STIM1R304W or Orai1P245L on hypocalcemia.

We show that overexpression of Gα11 increases TRPC1-mediated Ca^2+^ influx and Gα11 physically interacts with both the N- and C-terminus of TRPC1. We propose that TRPC1 can be directly activated by a physical interaction with Gα11. Our data do not favor the idea that freed Gβγ complex mediates an effect on TRPC1, because inhibition of Gβγ complex with Gallein did not affect PTH secretion in PTH-C1 cells (our unpublished observations). Direct Gαq-mediated regulation of TRP channels was first described in the *Drosophila* TRPL channel (31), which shows the closest homology to members of the TRPC subgroup of mammalian TRP channels. Recently, TRPC4 or TRPC5 was shown to physically interact with Gαq, which is structurally related to Gα11 (48). We have shown that TRP channels, including the canonical group (TRPC1-7), require an intramolecular interaction between their N- and C-termini during activation (49). This intramolecular interaction is influenced by levels of membrane phospholipid, PIP_2_, and mediated by a tryptophan residue in the pre-S1 domain and an arginine residue in the TRP-box domain in the C-terminal tail. It would be interesting to know whether interaction of TRCP1 and Gα11 involves these domains. Nevertheless, because Gα11 interacted with both the N- and C-terminus of TRPC1 and overexpression of Gα11 increased TRPC1 activity, we speculated that a physical interaction with Gα11, following PIP_2_ breakdown would stabilize the N-/C-interaction causing faster activation of TRPC1.

In summary, our data identify TRPC1 as one of the channels mediating a critical step in the suppression of PTH secretion in response to a rise in serum Ca^2+^. Information generated by our studies could be useful in designing new and more effective therapeutic approaches for diseases of the PTG by targeting molecules downstream of CaSR.

## Methods

### Mice

Mice were maintained under pathogen-free condition in the barrier facility of University of Oklahoma Health Sciences Center. WT (*Trpc1*^+/+^) and *Trpc1*^−/−^ mice were on a pure 129/SvEv background (19).

### Cell culture

PTH-C1 cells, a cell line derived from rat PTG, were a gift from Maria Luisa Brandi (FirmoLab, Università degli Studi di Firenze) and cultured in prescribed conditions (26). HEK293 and HEK293T cells were purchased from ATCC and cultured in DMEM (Corning; 10-013) enriched by 10% FBS (Atlanta Biologicals; S11550).

### In vivo studies

#### Collection of blood, urine, and clearance samples.

Mice were weighed every 7–10 days. Studies were performed on mutant (*Trpc1^–/–^*) and littermate controls (*Trpc1^+/+^*) from 3.5 to 21.5 months of age. Twenty-four-hour urine was collected at various times over 21.5 months in individual metabolic cages. A trace amount of sugar was added to drinking water to promote ingestion and to increase urine volume in order to optimize the completeness of collection, as verified by daily creatinine excretion. Water and food intake was monitored during the balance studies. Blood was collected by tail bleed, fasted or unfasted, whichever appropriate, periodically from age 7 to 21.5 months, usually synchronized with metabolic urine collections, as appropriate. Renal clearance was performed in individual mice at 21.5 months to measure Ca^2+^ in urine directly collected from urinary bladder via indwelling catheters and to measure glomerular filtration rate by intravenously infused inulin using methods we established, as described earlier (50).

### Measurements of creatinine, Ca^2+^, and Mg^2+^ in urine and blood and analysis of blood PTH, calcitriol, and calcitonin

Creatinine was measured by HPLC (Buck Scientific). Ca^2+^ was measured using Ca Arsenazo III dye adapted for a plate reader (Pointe Scientific). Mg^2+^ was measured using Arsenazo dye (Thermo Scientific Fisher). PTH was measured using a Mouse PTH 1-84 ELISA kit (Immunotopics). Mouse calcitriol (or 1,25-dihydroxyvitamin D) and calcitonin were measured using ELISA kits (Mybiosource, catalog MBS816417 and MBS026850, respectively).

### RNA interference

PTH-C1 cells were cultured to 70% confluency and transfected with siTRPC1 (Dharmacon; L-080128-02-0005) or siOrai1 (Dharmacon; L-081151-02-0005) or nontargeting siRNA (Dharmacon; D-001810-10-05) using Lipofectamine3000 (Invitrogen). Two days after transfection, cells were transferred into 24-well plates for PTH secretion analysis or 18-mm glass disks for single-cell Ca^2+^ imaging analysis.

### CRISPR/Cas9

Exon 1 of the rat *Trpc1* locus was edited using CRISPR/Cas9 and the following guide: (T2) 5′-CACCGGGCGCTGAAGGATGTGCGAG-3′ or (T3) 5′-CACCGGGCGGCCCTGTACCCGAGCA-3′. The *Trpc1*-specific sgRNA (T2) was cloned into lentiCRISPRv2 puro vector (Addgene) and used to transfect PTH-C1 cells. Puromycin-resistant stable clones were obtained and expanded, and gene editing of the *Trpc1* locus was determined by Sanger sequencing.

### Patients and DNA sequence analysis

Nineteen unrelated patients with FHH in whom previous mutational analysis of CASR, GNA11, and AP2S1 genes by Sanger DNA sequencing had not identified any abnormalities of the coding regions and exon-intron boundaries, were analyzed. DNA sequence analyses, using Sanger sequencing, of TRPC1 exons 1–12 of transcript ENST00000273482 and their adjacent splice sites were performed using leucocyte DNA and gene-specific primers (MilliporeSigma) (Supplemental Table 1), as previously reported (24). WES was performed using leucocyte DNA, as previously described (4). Publicly accessible databases were examined for the presence of sequence variants, including dbSNP (http://www.ncbi.nlm.nih.gov/projects/SNP/); 1000 genomes (https://www.internationalgenome.org/home); the National Heart, Lung and Blood Institute Exome Sequencing Project (http://evs.gs.washington.edu/EVS/, EVS data release ESP6500SI), representing the exomes of approximately 6500 individuals; and the gnomAD v2.1.1 database (https://gnomad.broadinstitute.org/), representing 125,748 exomes and 15,708 genomes of unrelated individuals mapped to the GRCh37/hg19 reference sequence.

### Expression plasmids

Mouse PTH (MR200486) and Gnα11 (MR205495) cDNAs were purchased from Origene. Human Orai1 (BC015369), mouse STIM1 (BC021644), and mouse TRPC1α or TRPC1ε (CA327829) were obtained from Open Biosystems, as described earlier (23). TRPM4 and TRPM7 cDNAs were obtained from A. Scharenberg (University of Washington, Seattle, Washington, USA).

### Single cell Ca^2+^ imaging

#### Fura2/AM labeling.

PTH-C1 cells were plated onto glass coverslips and loaded with 2 μM Fura-2/AM in extracellular solution (ECS) containing 140 mM NaCl, 5 mM KCl, 1 mM MgCl_2_, 1.8 mM CaCl_2_, 10 mM glucose, and 15 mM HEPES, pH 7.4 ([Ca^2+^]_o_: 1.8 mM) in the presence of 0.05% Pluronic F-127 (ThermoFisher catalog P3000MP) for 45 minutes at room temperature. Cells were washed twice in ECS and incubated for 15 minutes in 37°C before intracellular imaging. Cells were incubated in ECS (Figure 5, B and C) or a Ca^2+^-free solution (same as ECS but without CaCl_2_) (Figure 5D) and stimulated with extracellular Ca^2+^ (Figure 5B) or spermine (Figure 5, C and D) at the indicated times. Individual cells were excited by the DeltaRam X monochromator (Photon Technology International), and emission images were collected by a high-definition imaging scientific CMOS camera driven by the EasyRatioPro software (Photon Technology International). Fluorescence ratios of 340/380 were taken every 5 seconds using a 30-millisecond exposure time. Intracellular Ca^2+^ concentration was expressed as 340/380 ratio.

#### GCaMP3 labeling.

PTH-C1 (Figure 6E) or HEK293 cells (Figure 3F; Figure 5, F and G; and Figure 6, A and B) were transfected with GFP-based calcium sensor for imaging calcium dynamics (GCaMP3). GCaMP3 was a gift from Loren Looger (Addgene plasmid, catalog 22692). Two days after transfection, cells were processed for single-cell Ca^2+^ imaging as described for Fura2/AM-labeled cells, but excitation was set at 474 nm and emission at 510 nm. Fluorescence intensity was acquired every 5 seconds for 100 milliseconds, and intracellular Ca^2+^ concentration was expressed as the ratio of fluorescence signal at any given time point over baseline fluorescence (F_0_, average fluorescence intensity before the addition of drug) (F/F_0_).

### Ex vivo secretion of PTH

Sixteen-week-old mice were euthanized and PTGs were extracted. PTH secretion was performed as previously described (23). Glands were immediately transferred into 500 μl inhibition buffer solution (3 mM Ca^2+^, 0.5 mM Mg^2+^, 0.2% BSA, 20 mM HEPES/MEM-EBSS-CMF, pH 7.4) and kept on ice. Next, glands were transferred onto 0.1 μM Nucleopore Track-Etch Membrane (Whatman; 110405) and equilibrated in inhibition solution for 1 hour in a 37°C/5% CO_2_ incubator. Membranes with glands were transferred in buffer solutions (500 μl) containing 0.5, 0.75, 1, 1.25, 1.5, 2, or 3 mM Ca^2+^ for 30 minutes. Secreted PTH was determined using the Mouse PTH 1-84 ELISA Kit (Immutopics; 60-2305).

### In vitro secretion of PTH

Secreted PTH from native and transiently or stably transfected PTH-C1 cells was determined using a Rat Intact PTH ELISA Kit (Immutopics; 60-2500). PTH was collected after 4-hour incubation in fresh secretion media solution (0.5 mM Ca^2+^, 0.5 mM Mg^2+^, 0.2% BSA, 20 mM HEPES/MEM-EBSS-CMF, pH 7.4) prior to quantification of PTH.

### PCR

PTH-C1 cells were collected in TRIzol (Invitrogen; 15596026). RNA isolation was performed according to the manufacturer’s instructions. Five μg RNA was used for reverse transcription. mRNA was combined with oligo (dT) 12-18 primer (Invitrogen; 18418012) and random hexamers (Invitrogen; N8080127) and incubated for 10 minutes in 70°C. Next, 5× buffer, 0.5 μM dNTP, 10 mM DTT, and 20 U/μl SuperScript III were added (reverse transcription kit 18080093 from Invitrogen) and reaction was performed under the following conditions: 42°C for 45 minutes, 52°C for 30 minutes, and 70°C for 15 minutes. Primers used for further PCR were as follows: TRPC1 forward, GAGTTACCTTCGGCTCTTCTTT, reverse, GCTGAGGCTGCTGATCATATAG; nested forward, GCTCTGTTCTGGTACATCTTCTC, reverse, GGCAGTGTGCATTTGTCATC; TRPC4 forward, GAATGCTCCTGGACATCCTAAA, reverse, CCTCATCACCTCTTGGTATTGG; nested forward, GGTTAAGCTGCAAAGGCATAC, reverse, CCAAAGCTTTCTGGCTTTCTTC; TRPC5 forward, CAAGGTCCCGACTGAACATATAC, reverse, GCATGAAGAGGAAGGTCAGATAG; and nested forward, CTTCGCTCATCGCCTTATCA, reverse, ATGCTGTGTGGCAGATGAA.

### Affymetrix Mouse Microarray analyses

For RNA expression in mouse PTGs, 4 batches of glands (PTGs) were dissected free of thyroid and the surrounding fibrous tissues and used for RNA extraction with a RNA-Stat 60 kit (Thermo Fisher Scientific) as described previously (25). The RNA was reversed transcribed into cDNA, which was then subjected to Affymetrix Mouse GeneChip Microarray analyses by the Genome Technologies Core Facility (University of Manchester) and Genome Analyses Microarray Core (University of California, San Francisco). The gene array data were analyzed using Affymetrix Genechip Software for an intensity value and normalized and presented as a percentage of the expression level of a mitochondrial microsomal protein L19 and used for statistical analyses (*n* = 4 batches of RNA with each batch extracted from 20 PTGs dissected from 10 of 6-week-old C57bB6 mice).

### Coimmunoprecipitation

PTH-C1 or HEK293T cells were lysed in native lysis solution (1% Triton X-100, 150 mM NaCl, 10 mM Tris-HCl, pH. 7.5, 1 mM EDTA, 1 mM EGTA, 0.5% Igepal, 10 % sucrose, 5 mM NaF, 200 μM vanadate, protease inhibitor) in 4°C for 30 minutes. Cleared lysates were collected and used for coimmunoprecipitations. Myc- or GST-tagged proteins were captured using a rabbit monoclonal antibody against myc tag (clone 71D10, Cell Signaling; 2278) or Glutathione Sepharose 4B (GE Healthcare; GE17-0756-01), respectively. TRPC1 was detected using 1F1 (51), GαS (Santa Cruz; sc-135914), or GST (Santa Cruz; sc-459). Gα11 was detected using FLAG antibody (clone M2, MilliporeSigma; F1804).

### Immunohistochemistry staining for mouse PTG

Four-micron-thick histological sections, embedded in paraffin and mounted on HistoBond Plus slides (Statlab Medical Products), were rehydrated and washed in Tris-buffered saline. The sections were processed for immunohistochemistry using M.O.M. (Mouse on Mouse ImmPRESS Peroxidase Polymer kit, Vector Labs) or, for rabbit antibodies, the ImmPRESS –VR Horse Anti-Rabbit IgG Polymer Peroxidase kit (Vector Labs). Antigen retrieval (pH 6 Citrate Antigen Unmasking Solution, Vector Labs) was accomplished via 20 minutes in a steamer followed by 30 minutes cooling at room temperature. Sections were treated with a peroxidase blocking reagent (Bloxall, Vector Laboratories)

### Double indirect immunofluorescence labeling

PTH-C1 and PTH-C1*^Trpc1-KO^* cells seeded on coverslips were fixed with cold methanol for 5 minutes in room temperature and then washed 3 times with ice-cold PBS. Cells were permeabilized with 0.5% Saponin solution for 10 minutes followed by 3 washes in PBS. Blocking was done with 3% BSA for 15 minutes, and then cells were incubated with primary antibody (TRPC1-1F1, 1:500, Gα11 1:200) diluted in 1% BSA in 4° C overnight. Secondary antibodies coupled to Alexa Fluor 488 (Thermo Scientific Fisher; A11029, used at 1:2,000 dilution) or Alexa Fluor 594 (Thermo Scientific Fisher; A11012, used at 1:2,000 dilution) were added on coverslips for 2 hours at 4°C followed by 3 washes in PBS. Coverslips were mounted with Diamond DAPI solution (ProLong Diamond Antifade Mountant with DAPI, Thermo Scientific Fisher; P36962). Images were acquired and processed with laser scanning confocal microscope (Olympus Fluoview 1000) in an inverted configuration.

### Statistics

All experiments showing protein-protein interactions and indirect immunofluorescence staining were repeated at least 3 times. Data measurements were presented as mean value ± SEM. Differences between 2 groups were determined by unpaired, 2-tailed Student’s *t* test or Mann Whitney test (if data within groups fail to show normal distribution as determined by the D’Agostino-Pearson normality test). Significant differences between more than 2 groups were determined by 1- or 2-way ANOVA, as indicated followed by Sidak’s multiple comparison test or Kruskal-Wallis test followed by Dunn’s multiple comparison test (if data failed the D’Agostino & Pearson normality test). All statistical analyses were performed using GraphPad Prism 7 software. *P* values of less than 0.05 were considered significant.

To establish whether our analysis was sufficiently powered to detect at least 1 *TRPC1* mutation with a greater than 95% likelihood, the sample size required was determined by binomial probability analysis (Microsoft Excel), as previously reported (24). Approximately 65% of patients with FHH1 have a *CASR* mutation (52), and for this binomial analysis the prevalence for *TRPC1* mutations in patients with FHH without *CASR*, *GNA11*, or *AP2S1* mutations was set at 20%, and a similar approach to that described for a search of *AP2S1* mutations in ADH patients was used (24). The binomial distribution probability was calculated using the following formula: binomial distribution probability = *b*(*x*;*n*,*p*), where *b*(*x*;*n*,*p*) = (*nx*)*px*(1−*p*)*n*−*x*, where *n* indicates sample size, *x* represents the number of probands harboring mutations; *n*–*x* denotes the number of probands with no mutation; and *p* represents the prevalence of *TRPC1* mutations in the cohort.

### Study approval

All procedures were approved by the IACUC of University of Oklahoma Health Sciences Center (301163 OUHSC IACUC original protocol approval_18-101_11/7/2018#18-101). Informed consent was obtained from individuals using protocols approved by the multicentre research ethics committee (MREC), London, United Kingdom, (approval code MREC/02/2/93).

## Author contributions

MO, VN, and LT performed and analyzed in vitro experiments. MO, PN, BE, and KL performed and analyzed in vivo experiments. MO, MBH, and WC performed ex vivo experiments. ML performed immunohistochemistry staining studies. MLB provided critical reagents. CMG, VJS, and RVT acquired and analyzed human DNA-sequencing data. MO and LT wrote the manuscript with the help of MBH and KL. LT oversaw the project.

## Supplementary Material

Supplemental data

## Figures and Tables

**Table 1:**
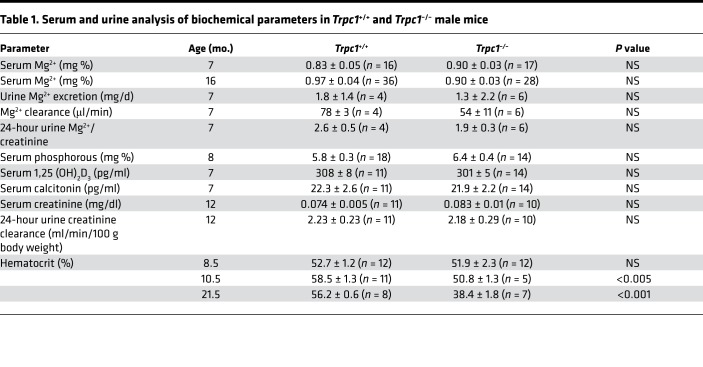
Serum and urine analysis of biochemical parameters in *Trpc1*^+/+^ and *Trpc1*^–/–^ male mice

## Version 1 03/26/2020

In-Press Preview

## Version 2 04/23/2020

Electronic publication
