# Supplementary material for: Control of PTH secretion by the TRPC1 ion channel
Source: JCI Insight. 2020 Apr 23;5(8):e132496. doi: 10.1172/jci.insight.132496 (PMC7205425; doi:10.1172/jci.insight.132496)
Supplement: Supplemental data [file jciinsight-5-132496-s096.pdf]

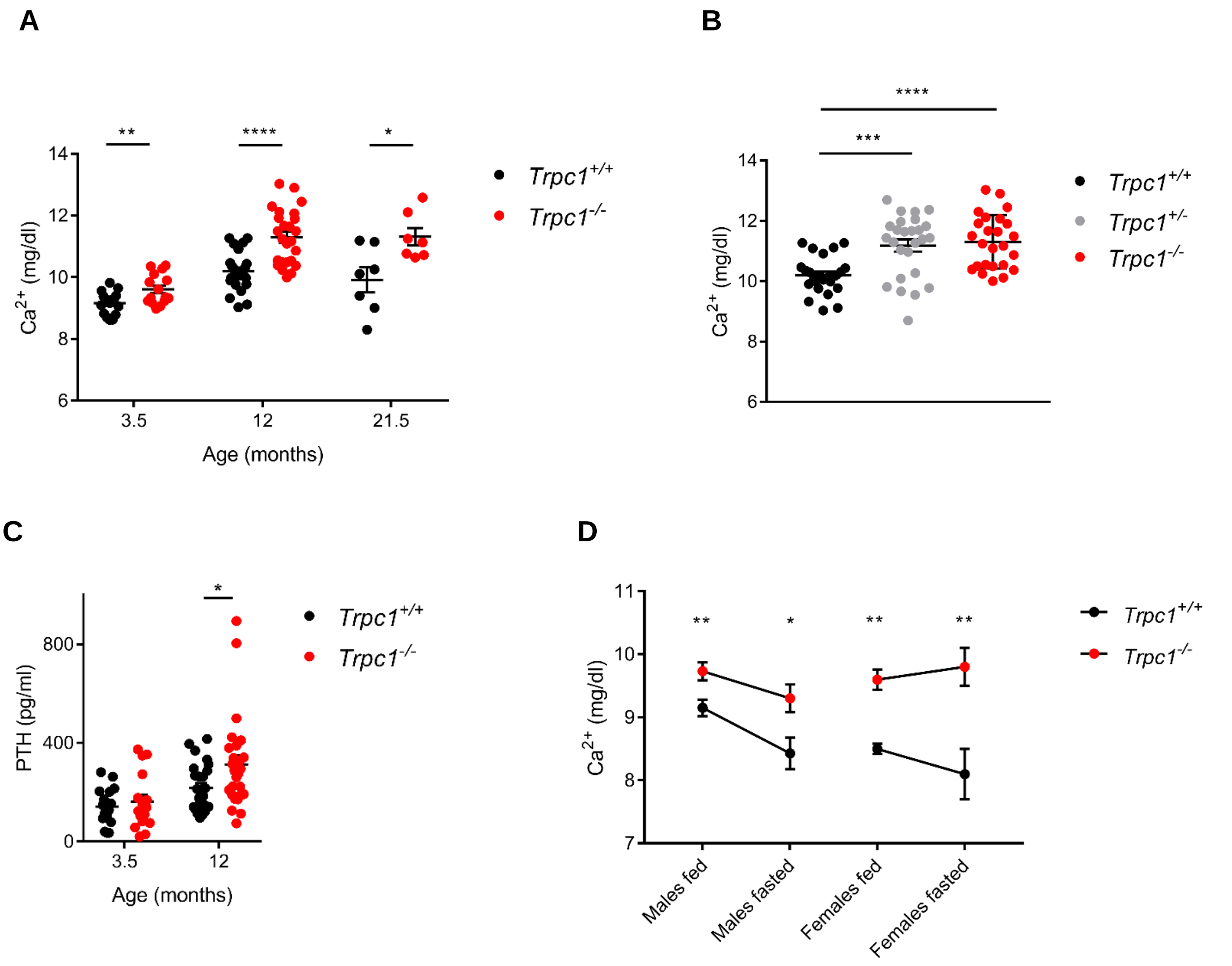

Figure S1 ***Trpc1***$^{-/-}$ **mice exhibit hypercalcemia independently of age, heterozygous deletion or fasting.**

A) Serum Ca^{2+} levels (mg/dl) in 3.5, 12 and 21.5 month-old *Trpc1*$^{+/+}$ and *Trpc1*$^{-/-}$ unfasted males. *, p<0.05; **, p<0.01; ****, p<0.0001 Student's t test.

B) Ca^{2+} levels (mg/dl) in 12 month-old *Trpc1*$^{+/+}$, *Trpc1*$^{+/-}$ and *Trpc1*$^{-/-}$ fasted male mice. ***, p<0.001; ****, p<0.0001, one-way ANOVA.

C) Serum PTH levels (pg/ml) in 3.5 and 12 month-old unfasted male *Trpc1*$^{+/+}$ and *Trpc1*$^{-/-}$ mice. *, p<0.05, Student's t test.

D) Serum Ca^{2+} levels (mg/dl) in 7 month-old *Trpc1*$^{+/+}$ and *Trpc1*$^{-/-}$ freely fed and fasted male and female mice. *, p<0.05; **, p<0.01, Student's t test.

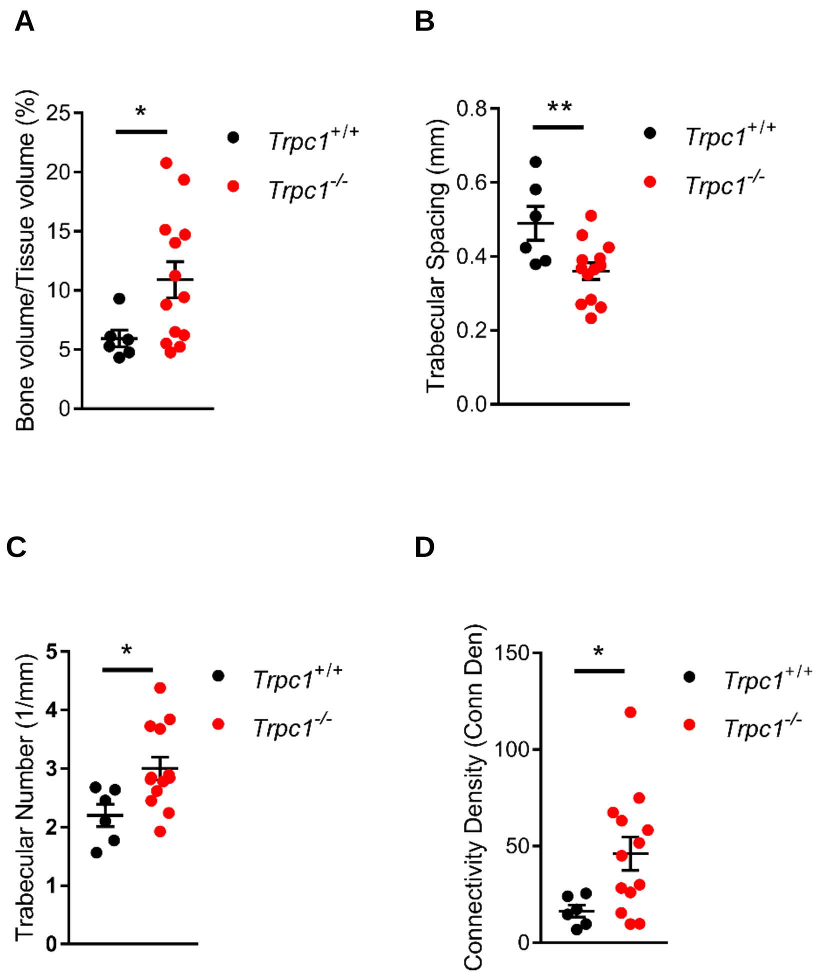

Figure S2 *Trpc1*$^{-/-}$ mice exhibit increased bone mass

A) Bone volume/tissue volume (BV/TV) (%) in 19 month-old *Trpc1*$^{+/+}$ (n=6) and *Trpc1*$^{-/-}$ (n=13) males, determined by μCT.
B) Trabecular spacing (Tb Sp) (mm) in 19 month-old *Trpc1*$^{+/+}$ (n=6) and *Trpc1*$^{-/-}$ (n=13) males, determined by μCT.
C) Trabecular number (Tb N) (1/mm) in 19 month-old *Trpc1*$^{+/+}$ (n=6) and *Trpc1*$^{-/-}$ (n=13) males, determined by μCT.
D) Connectivity density (conn den) in 19 month-old *Trpc1*$^{+/+}$ (n=6) and *Trpc1*$^{-/-}$ (n=13) males, determined by μCT.

*,p<0.05; **, p<0.01. Student's t test.

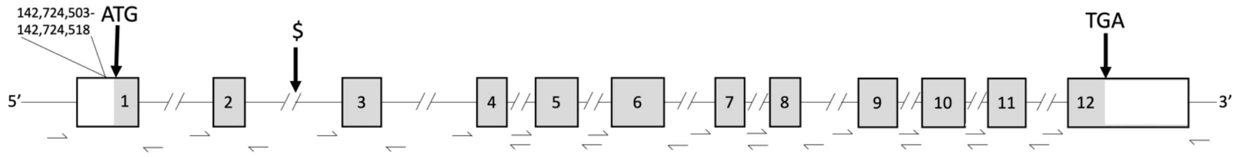

Figure S3 Schematic representation of the human TRPC1 transcript

Human *TRPC1* transcript ENST00000273482 encoding the short 759 amino acid isoform, which expression studies (data obtained from the GTEx portal on 24/02/2020 and dbGaP Accession phs000424.v8.p2) indicate is expressed at much higher (>10 fold) levels in all tissues than the longer isoform (793 amino acids) encoded from transcript ENST00000476941 that contains an additional exon 3 (position indicated by $). Coding regions are shaded grey and untranslated regions (UTRs) are represented by open boxes. Exons 1 and 12 contain the 5' and 3' UTRs, respectively. Exon 1, containing the ATG start codon (A position 142,724,561; GRCh38.p13), comprises 563 nucleotides of which 391 nucleotides are the 5'UTR and 172 nucleotides are coding sequence. DNA sequence analysis, using Sanger sequencing or WES, of leucocyte DNA from 19 FHH patients, did not detect any abnormalities in: the 12 coding exons encoded by transcript ENST00000273382, or within their intron-exon boundaries; the 391bp of the 5'UTR in exon1; and the entire 3'UTR. However, a 16 nucleotides segment at position 142,724,503-142,724,518 (indicated at the top of the transcript map) of the 5'UTR contains repetitive elements, and the DNA sequence of this short non-coding region could not be ascertained reliably. In addition, the additional exon 3 encoded from transcript ENST00000476941, which has a low expression, could not be determined by Sanger sequencing or WES. The locations of the DNA primers used to amplify each exon and associated intron-exon boundaries are indicated below each exon.

A

PTH-C1 GGCGCTGAAGGATGTGCGAG**AGG**TGAAGGAGGAGAAC

PTH-C1$^{\mathit{Trpc1}\text{-KO}}$

GGCGCTGAAGGATGT–CGAGAGGTGAAGGAGGAGAAC
GGCGCTGAAGGATGTG**C**CGAGAGGTGAAGGAGGAGAAC
GGCGCTGAAGGATGT–CGAGAGGTGAAGGAGGAGAAC
GGCGCTGAAGGATGT–CGAGAGGTGAAGGAGGAGAAC
GGCGCTGAAGGATGT–CGAGAGGTGAAGGAGGAGAAC
GGCGCTGAAGGATGTG**C**CGAGAGGTGAAGGAGGAGAAC
GGCGCTGAAGGATGT–CGAGAGGTGAAGGAGGAGAAC
GGCGCTGAAGGATGT–CGAGAGGTGAAGGAGGAGAAC
GGCGCTGAAGGATGT–**A**GAGAGGTGAAGGAGGAGAAC
GGCGCTGAAGGATGTG**C**CGAGAGGTGAAGGAGGAGAAC
GGCGCTGA**G**GGATGT–CGAGAGGTGAAGGAGGAGAAC
GGCGCTGAAGGATGT–CGAGAGGTGAAGGAGGAGAAC
GGCGCTGAAG – – – – – – – – GAGGTGAAGGAGGAGAAC
GGCGCTGAAGGATGTG**C**CGAGAGGTGAAGGAGGAGAAC

B

PTH-C1$^{\mathit{Pth}\text{-1}}$ GGCGCTGAAGGATGTGCGAG**AGG**TGAAGGAGGAGAAC

PTH-C1$^{\mathit{Pth}\text{-1}\ \mathit{Trpc1}\text{-KO}}$

GGCGCTGAAGGATGT**CC**CGAGAGGTGAAGGAGGAGAAC
GGCGCTGAAGGATGTG**C**CGAGAGGTGAAGGAGGAGAAC
GGCGCTGAAGGATGTG**C**CGAGAGGTGAAGGAGGAGAAC
GGCGCTGAAGGATGTG**C**CGAGAGGTGAAGGAGGAGAAC
GGCGCTGAAGGA – – – – – – – – – – – – – – – – – – – GAAC
– –
GGCGCTGAAGGATGTG**GATGAGAAGGAT**GAGAGGTGAAGGAGGAGAAC
GGCGCTGAAGGA – – – – – – – – – – – – – – – – – – – GAAC
GGCGCTGAAGGA – – – – – – – – – – – – – – – – – – – GAAC
GGCGCTGAAGGA – – – – – – – – – – – – – – – – GGAGAAC
GGCGCTGAAGGATGTG**GATGAGAAGGAT**GAGAGGTGAAGGAGGAGAAC
GGCGCTGAAGGATGTG**C**CGAGAGGTGAAGGAGGAGAAC
GGCGCTGAAGGATGTG**C**CGAGAGGTGAAGGAGGAGAAC

C

PTH-C1$^{Pth\text{-}2}$	GGCGCTGAAGGATGTGCGAG**AGG**TGAAGGAGGAGAAC
PTH-C1$^{Pth\text{-}2\ Trpc1\text{-}KO}$	GGCGCTGAAGGA ---------------- GGAGAAC
	GGCGCTGAAGGA ---------------- GGAGAAC
	GGCGCTGGAGGATGTG**C**CGAGAGGTGAAGGAGGAGAAC
	GGCGCTGAAGGATGTG**C**CGAGAGGTGAAGGAGGAGAAC
	GGCGCTGAAGGA ---------------- GGAGAAC
	GGCGCTGAAGGA ---------------- GGAGAAC
	GGCGCTGAAGGATGTG**C**CGAGAGGTGAAGGAGGAGAAC
	GGCGCTGAAGGA ---------------- GGAGAAC
	GGCGCTGAAGGATGTG -------- AAGGAGGAGAAC
	GGCGCTGAAGGATGTG-**A**AGAGGTGAAGGAGGAGAAC
	GGCGCTGAAGGATGTG**C**CGAGAGGTGAAGGAGGAGAAC
	GGCGCTGAAGGA ---------------- GGAGAAC

D

PTH-C1 PTH-C1$^{Trpc1\text{-}KO}$

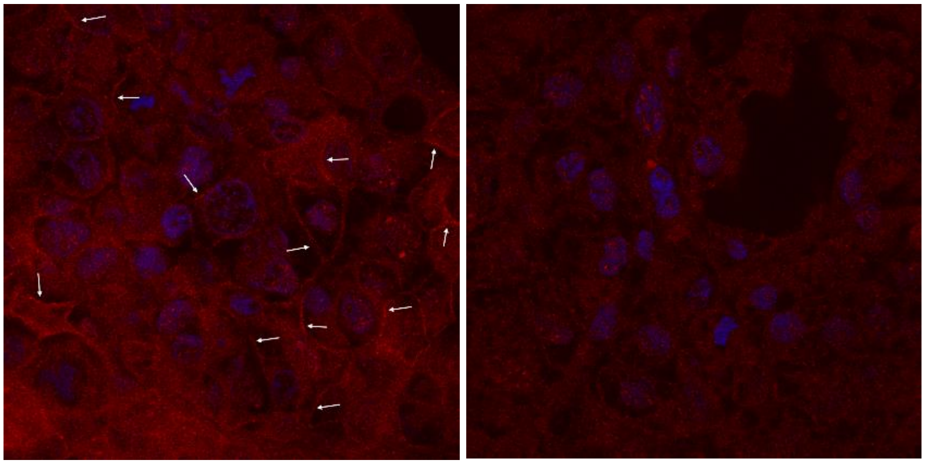

E

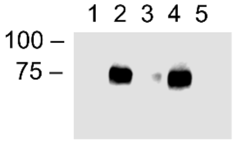

Figure S4 CRISPR/Cas9-mediated gene editing of the *Trpc1* locus in PTH-C1 cells.

A) Sequencing analysis around the recombination site followed by the PAM sequence (bold) in Exon 1 of wild type *Trpc1* (PTH-C1 cells) and PTH-C1$^{Trpc1\text{-}KO}$ cells. Sequences from 14 individual bacterial clones transformed with plasmids containing a PCR fragment of the recombination site in PTH-C1$^{Trpc1\text{-}KO}$ cells. No wild-type sequence was identified.

B) Sequencing analysis around the recombination site followed by the PAM sequence (bold) in Exon 1 of wild type *Trpc1* (PTH-C1 cells) and PTH-C1$^{Pth\text{-}1/Trpc1\text{-}KO}$ cells. Sequences from 13 individual bacterial clones transformed with plasmids containing a PCR fragment of the recombination site in PTH-C1$^{Pth\text{-}1/Trpc1\text{-}KO}$ cells. No wild-type sequence was identified.

C) Sequencing analysis around the recombination site followed by the PAM sequence (bold) in Exon 1 of wild type *Trpc1* (PTH-C1 cells) and PTH-C1$^{Pth\text{-}2/Trpc1\text{-}KO}$ cells. Sequences from 12 individual bacterial clones transformed with plasmids containing a PCR fragment of the recombination site in PTH-C1$^{Pth\text{-}2/Trpc1\text{-}KO}$ cells. No wild-type sequence was identified.

D) TRPC1 expression in PTH-C1 cells and PTH-C1$^{Trpc1\text{-}KO}$. Cells were stained with 1F1 antibody (red) and DAPI (blue). White arrows indicate cell membrane staining. Magnification: 100X; n=3.

E) Efficiency of knockdown of rat TRPC1 cDNA by CRISPR/Cas9 gene editing. HEK293T cells were mock-transfected (lane 1) or transiently co-transfected with rat TRPC1 cDNA plus empty vector control (lane 2), rat TRPC1 cDNA plus a rat TRPC1-specific short guided RNA (T2 sgRNA), rat TRPC1 cDNA plus a rat TRPC1-specific short guided RNA (T3 sgRNA) (lane 4) or rat TRPC1 cDNA plus rat TRPC1-specific short guided RNAs T2 and T3 (lane 5). Cells were lysed and expression of TRPC1 was determined by immunoblotting using a TRPC1-specific monoclonal antibody (1F1). T2, but not T3 sgRNA knocked down TRPC1.

A B

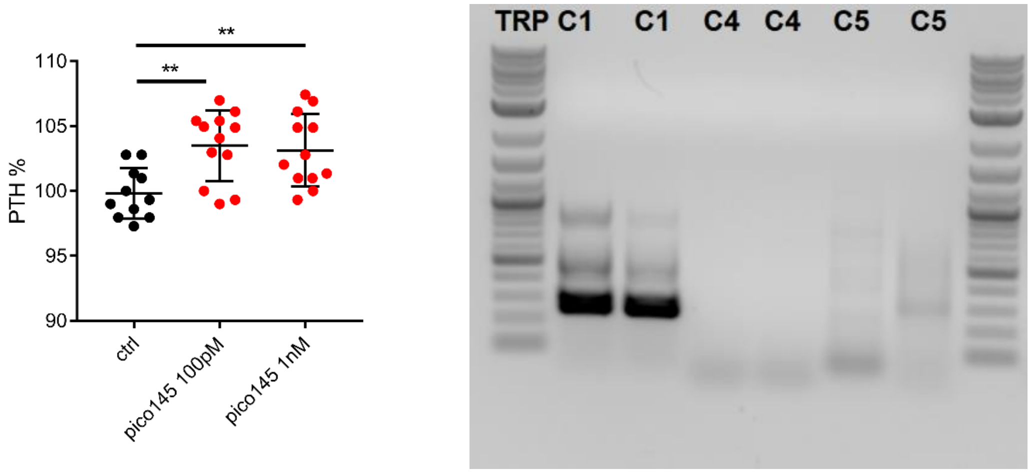

Figure S5 Pico145 increases PTH secretion in PTH-C1 cells

A) Effect of 100 pM or 1 nM pico145 on PTH secretion in PTH-C1 cells cultured in 2.5 mM of extracellular Ca^{2+}. Data represent 9-12 measurements pooled from 3 independent experiments. **, p<0.01. One-way ANOVA.

B) TRPC4 (352 bp) or TRPC5 (328 bp) are undetectable in PTH-C1 cells as determined by RT-PCR.

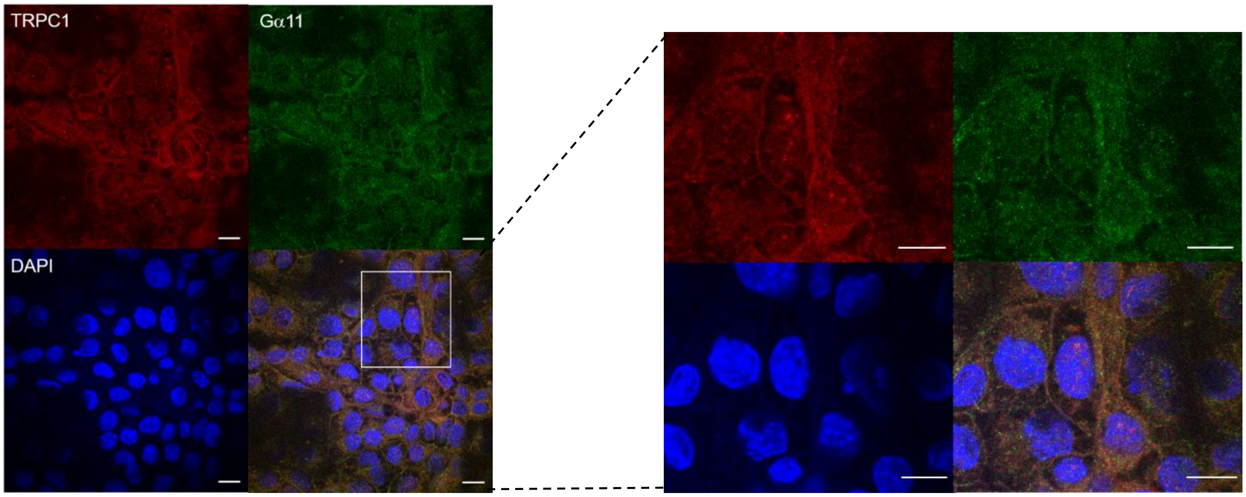

Figure S6 TRPC1 co-localizes with Gα11 in PTH-C1 cells.

Double Indirect immunofluorescence showing subcellular localization of Gα11 (green), TRPC1 (red), or together (yellow) in PTH-C1 cells. Nucleus is shown by DAPI staining (blue). Scale bar: 10 μm.

Exon	Forward Primer Sequence	Reverse Primer Sequence
**5'UTR**	5' AGAGTCGCGAACATCTCCTC	5' AGGAGAAAGGGGCCTAACTC
**1**	5' AGCAGTGGGAACGACTCATC	5' GAATGCCCCGATATAAGTTGA
**2**	5' TGTCTGCTGCTGATGTACGT	5' GAAGGGAAGAAAGCATGGCA
**3**	5' CATATTCCTTAAATAATTCTTA	5' CTTAAAAGCTGCATAGAA
**4**	5' AGCAATAGATAGCAAGGTTT	5' ACTGAGGTTGAAGATTTAAAACA
**5**	5' AGCTGTGGAAAATTCGTGCA	5' CCCTGAGCCACTAGACTACTG
**6**	5' GAATATCAACCAATCAGTA	5' CTTGTGGTGTGAACAGTGTA
**7**	5' TCTTTTGAATAAGGCCCAAGGA	5' AGACACTGATCTGGCTCAATG
**8**	5' TGGCTTTCTTTCAACAGTCAGA	5' ACAGTATTCCACTGGACAAGG
**9**	5' GATAATCTTAATGAACACCT	5' AGACAAATATATTTACTAAAAC
**10**	5' GATATAAACAAATGATTCA	5' AGTATGGTCTGAGGAGAATATA
**11**	5' TTTTCTGCAAGGTGTGGAAAC	5' AGACAGTCACAGCCCTTAGG
**12**	5' GAACTCTACCTCATTTAAAT	5' CAATATAGTCTTTTGGATCTG

Table S1 Primer sequences used for Sanger sequencing of *TRPC1* in 14 FHH patients. Exon numbering correspond to those in transcript ENST00000273482 encoding the shorter TRPC1 isoform.
